# Optimization of Culture Conditions for Human Ovarian Stem Cells Using Gelatin-Coated Surfaces

**DOI:** 10.7759/cureus.89628

**Published:** 2025-08-08

**Authors:** Sindhuja N Srinivasan, Sanjeeva Reddy, Alan M Punnoose, Mubeena S

**Affiliations:** 1 Reproductive Medicine and Surgery Department, Sri Ramachandra Institute of Higher Education and Research, Chennai, IND; 2 Stem Cell and Regenerative Biology Laboratory, Sri Ramachandra Institute of Higher Education and Research, Chennai, IND

**Keywords:** in vitro culture, isolation of stem cell, ovarian stem cell, ovary, stem cell, stem cell culture

## Abstract

Introduction: The presence of stem cells in the ovary has been a topic of discussion due to their questionable existence. Isolation of stem cells has been possible by enzymatic digestion; however, the percentage of cells harvested and expanded has not been satisfactory. This could be due to the lack of optimal adhesion provided by the standard commercial culture dishes, which affects the initial attachment and further growth of cells.

Methods: Ovarian tissue was collected during pelvic surgery after obtaining written informed consent from four patients. The tissue was exposed to enzymatic digestion using collagenase and hyaluronidase. The resulting cell suspension was cultured in a carbon dioxide incubator in petri dishes, which were either coated with gelatin or uncoated, and monitored serially for growth. The initial cells were counted and further passaged when confluent. The cell number and quality, along with embryoid body formation, were compared between the coated and uncoated dishes.

Result: The initial attachment of cells and further expansion were higher in the dishes that were coated with gelatin compared to the standard non-coated culture dishes.

Conclusion: The addition of gelatin to the culture dishes seems to be beneficial in the culture of stem cells isolated from the human ovarian tissue and their further expansion.

## Introduction

The diagnosis of primary ovarian insufficiency (POI) indicates the loss of the ovarian follicle pool; thus, fertility preservation interventions (such as oocyte, embryo, or ovarian tissue cryopreservation) would appear useful. However, the variable course of the condition, especially in its early course, indicates the potential for a window of opportunity for this approach. While this is advocated in reviews of the subject, there are no data available as to its success rates [1]. These considerations also apply to highly selected women with Turner syndrome (TS), who may have an opportunity during adolescence and early adulthood for fertility preservation treatments.

In most mammalian females, the ovaries are said to undergo age-related dysfunction and failure, which has historically been thought to occur as a consequence of the depletion of the non-renewable pool of female germ cells (primordial follicles) established before birth. This cornerstone of mammalian female reproductive biology was challenged by the work of Johnson et al., who suggested that mouse ovaries retain the capacity to produce germ cells throughout life [2].

The existence of stem/progenitor cells in adult mammalian ovaries has recently been confirmed, although significant confusion remains about the types of stem/progenitor cells present and their properties. The latest studies have shown that stem cells occur in most of the compartments of adult ovaries, from the ovarian surface epithelium and cortex to the follicles [3].

Human pluripotent stem (hPS) cells, including human embryonic stem cells (hES cells) and human-induced pluripotent stem cells (hiPS cells), can self-renew indefinitely while retaining the capacity to differentiate into any somatic cell type. They therefore have great potential in various applications, including basic developmental research, drug/toxicity screening, and cell-based therapeutics. The complex matrix requirements of hPS cells, which make up the hPS cell “niche,” are well documented, and traditionally, hPS cell expansion has necessitated culture on feeder cells and serum-containing media [4].

Cells are traditionally grown on tissue culture polystyrene (TCPS), which is low-cost, sterile, and semi-reusable, leading to its widespread use over other materials such as glass. TCPS undergoes rapid adsorption of proteins in biological fluid, creating a poorly defined surface for cell studies, where the identity, density, and orientation of the proteins are unknown [5].

However, incompatibility of these complex, ill-defined conditions with pharmacological and medical applications has driven the development of alternative strategies combining defined media with improved surfaces. Gelatin, a hydrolyzed form of collagen IV, is a component of the extracellular matrix (ECM) that can be used readily and at low cost. Other solutions typically include surface immobilization of cell-binding motifs, such as integrin-binding proteins, short peptides derived from vitronectin (VN) and laminin (LN), glycosaminoglycan (GAG)-binding peptides, and synthetic polymers [6,7].

Gelatin serves as a simpler, xeno-free, feeder-free alternative to these feeder layers by providing a basic adhesive surface that facilitates cell attachment and survival without directly influencing differentiation. Gelatin does not inherently induce differentiation, allowing cells to maintain expression of key pluripotency markers such as OCT4, NANOG, SOX2, and SSEA4, as long as the media composition is supportive. While gelatin does not actively contribute growth factors or ECM complexity like Matrigel, its inert, supportive nature makes it an excellent platform for feeder-free stem cell culture, provided that the media is sufficiently enriched to maintain pluripotency. Due to these advantages and its increasing use in stem cell applications, we undertook this study to evaluate the effectiveness of gelatin-coated culture surfaces in supporting the growth and stemness of ovarian stem cells under defined culture conditions.

## Materials and methods

This was a prospective study in which ovarian tissue samples were collected from four women undergoing laparotomy after obtaining written informed consent. The time taken from resourcing the ovarian tissue, its culture, and further passaging of these samples took one year (April 2019-May 2020). Ethical approval for the study was obtained from the Institutional Ethics Committee of Sri Ramachandra Institute of Higher Education and Research (approval number: NI/18/JAN/63/12) and the Institutional Committee of Stem Cell Research and Therapy (ICSCRT). An ovarian tissue sample was collected using a scalpel and transported to the laboratory in phosphate buffered saline (PBS). Each sample was weighed before processing and digestion.

Mechanical dissection

**Figure 1:**
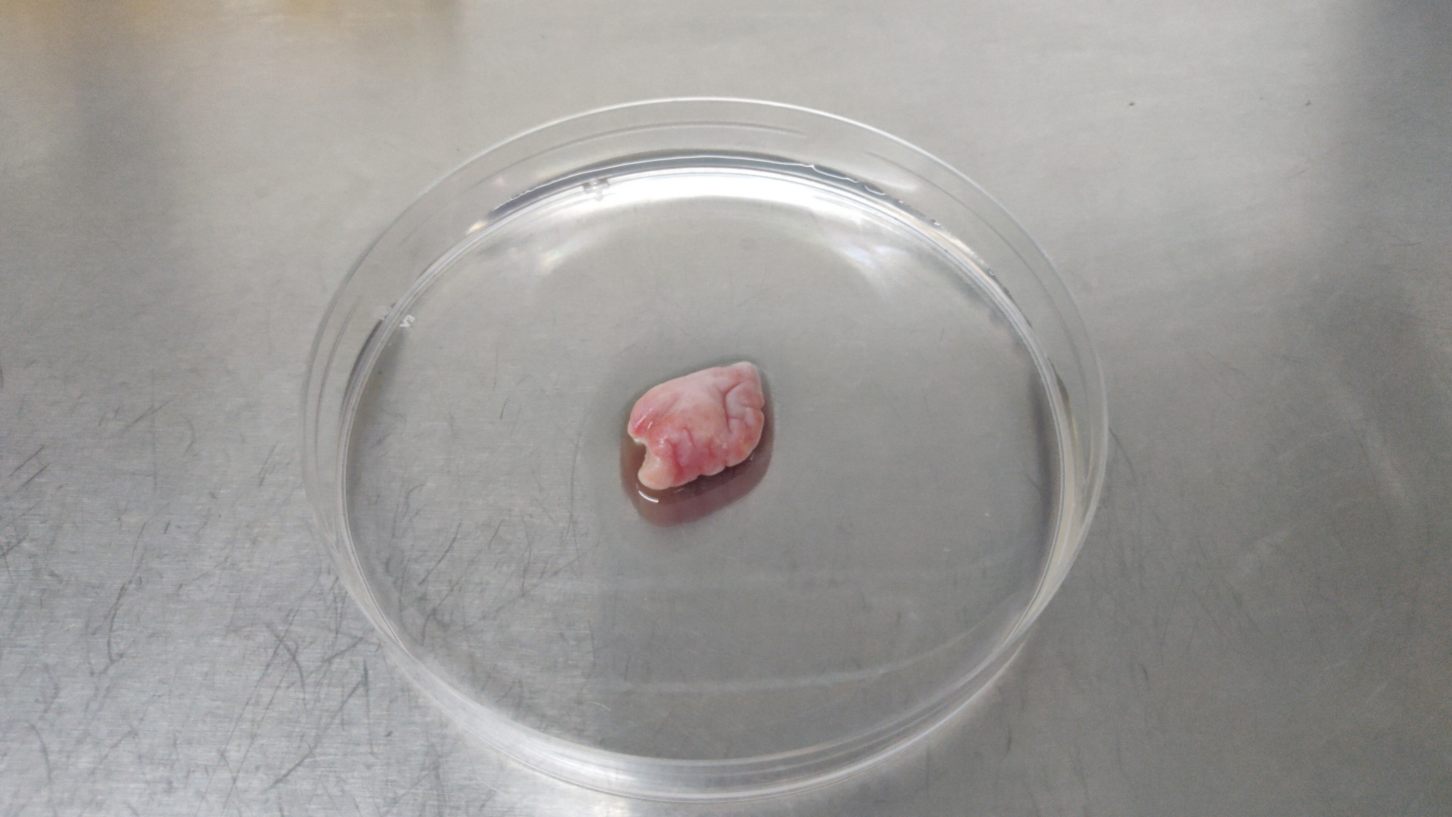
Ovarian cortical tissue before mechanical and enzymatic digestion

**Figure 2:**
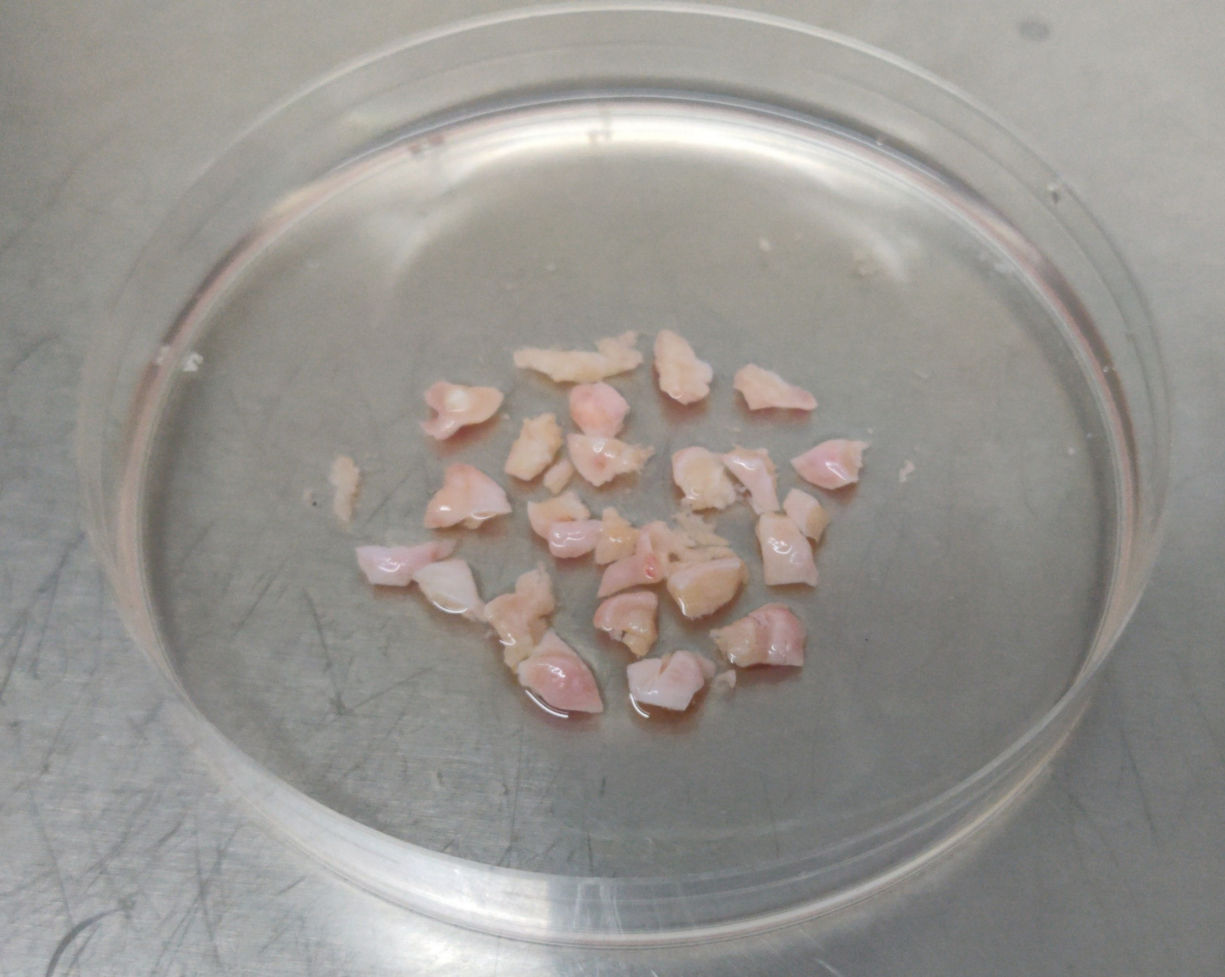
Ovarian tissue after mechanical dissection

The ovarian tissue samples were mechanically dissected with a scalpel with minimal media before exposing them to enzymatic digestion. Figure 1 and Figure 2 show the ovarian tissue before and after dissection, respectively.

Enzymatic digestion

**Figure 3:**
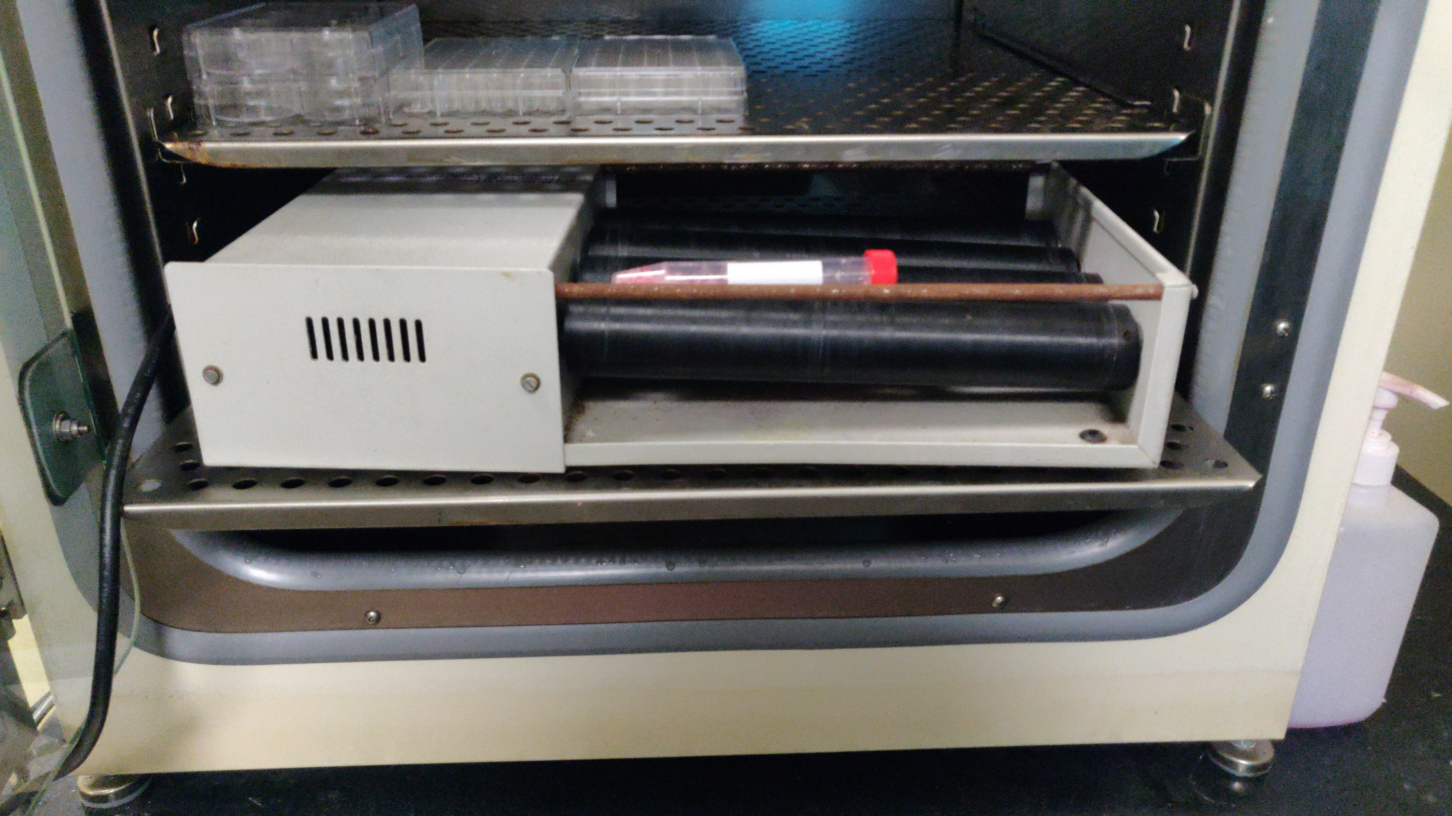
Enzymatic digestion on a blood roller mixer inside a carbon dioxide incubator

After dissection, digestion media composed of hyaluronidase enzyme (80 IU), collagenase enzyme (0.15% w/v), alpha minimal essential media (MEM), and L-glutamine was added to the tissue in a 50 mL conical tube and was placed in blood roller mixer inside a carbon dioxide incubator for one hour to overnight, with serial examination for digestion (Figure 3).

Preparation of culture media

The culture media for the cells was prepared by a mixture of alpha MEM, fetal bovine serum, and antibiotics, including penicillin, streptomycin, amphotericin, and ciprofloxacin.

Preparation of gelatin-coated dishes

A 2% (w/v) solution was prepared by dissolving gelatin (cell culture grade) (HiMedia Pvt. Ltd., Mumbai, India) in tissue culture grade water. Gelatin (2 mL) was added to 35 mm petri dishes and allowed to dry overnight. Excess gelatin was removed the next morning. Once dried, it was exposed to UV for 30 minutes and stored in the incubator with tight sealing until further use.

Cell culture

The digested cells were centrifuged and diluted with culture media and aliquoted on the culture dish with and without gelatin. Each dish was observed for initial attachment, cell growth, and expansion of cells in the gelatin-coated dishes and commercially available tissue culture dishes with periodic media changes under a Nikon inverted phase contrast microscope (Nikon, Tokyo, Japan).

## Results

The characteristics of the patients from whom the ovarian tissue sample was taken are mentioned in Table 1. Among the four women, two underwent laparotomy for total abdominal hysterectomy (TAH) with bilateral salpingo-oophorectomy (BSO), and two underwent lower segment caesarean section (LSCS) for delivering a baby.

**Table 1:** Overview of the four patients TAH: total abdominal hysterectomy, BSO: bilateral salpingo-oophorectomy, g: grams

Patient	Age (years)	Indication of surgery	Weight of tissue obtained
1	47	TAH + BSO	1 g
2	30	LSCS	0.3 g
3	32	LSCS	0.6 g
4	43	TAH + BSO	4 g

The outcome was measured in terms of initial cell attachment, cell expansion, and percentage of cells attached by cell counting using a Neubauer chamber in all four patients in both the gelatin-coated and commercial dishes using an inverted microscope (Nikon) at 20× magnification, and images were captured using software. The findings are tabulated in Table 2.

**Table 2:** Comparison of time taken for cell attachment, time taken for confluence, and percentage of cells attached between commercial dishes and gelatin-coated dishes among the four samples

	Sample 1		Sample 2		Sample 3		Sample 4	
	Commercial dish	Gelatin-coated dish	Commercial dish	Gelatin-coated dish	Commercial dish	Gelatin-coated dish	Commercial dish	Gelatin-coated dish
Time taken for initial attachment	48 hours	Within 4 hours	Did not attach	Within 4 hours	48 hours	Within 4 hours	Did not attach	Within 4 hours
Time taken for confluence	7 days	2 days	NA	2 days	10 days	2 days	NA	2 days
Percentage of cells attached	40%	80%	NA	90%	20%	80%	NA	80%

**Figure 4:**
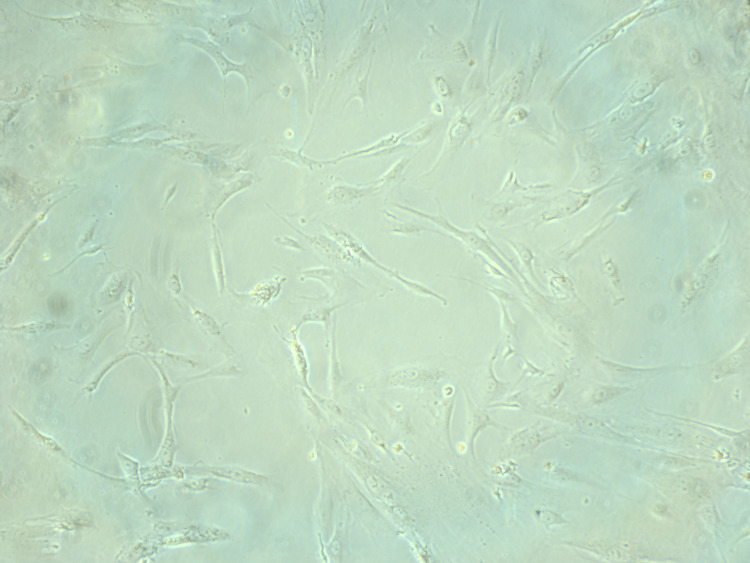
Initial attachment of cells in non-coated dishes

**Figure 5:**
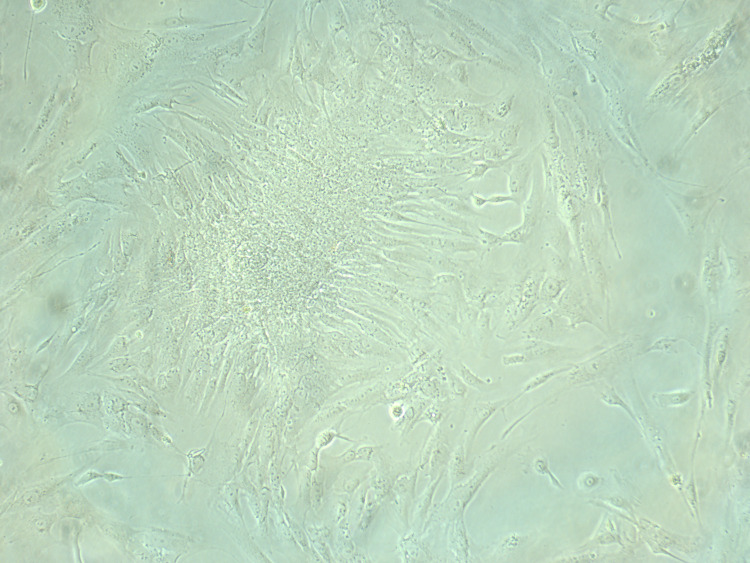
Initial attachment of cells in coated dishes

The number of cells attached after initial digestion was higher in the gelatin-coated dishes compared to the plain dishes, as shown in Figure 4 and Figure 5, taken on an inverted Nikon microscope with a camera software.

The further expansion of the cells attached was also more rapid in the gelatin-coated dish group compared to the plain dishes. This was observed by two different observers subsequently, in order to avoid any bias, and cells were counted with a cell counter and marked at areas of colony formation.

The dish reached a confluent state of culture only when the dishes were coated with gelatin; similarly, embryoid body formation and oocyte-like cells, which are seen in ovarian stem cell culture, were observed only in the coated dishes. Such cells and findings were not found to be seen in the non-coated dishes.

## Discussion

The importance of having gelatin-coated dishes is highlighted in this study, as it evidently helped in the rapid initial attachment of cells, maintenance of culture, and further propagation. Although commercially available dishes are suitable for stem cell culture, gelatin coating of dishes seems to be more beneficial in standardizing ovarian stem cell culture in vitro.

Regulation of stem cell behavior using two-dimensional synthetic templates in vitro is of immense importance in regenerative medicine [8]. Control over cell behaviors such as adhesion, proliferation, and differentiation may facilitate increased therapeutic applications of stem cells. Chemically defined growth of stem cells allows for quantifiable cell material interactions. It is highly desirable to have a chemically defined coating that is compatible with multiple substrate types and is stable over the long term in cell culture conditions.

This protocol is simple and easy to implement. However, some steps are critical to its success. The most important step is the aseptic isolation of stem cells from the ovarian cortex. Owing to the collection methods for the ovarian tissue, the contamination risk of pathogens is high. All of the procedures, including the coating of dishes, processing of the tissue, and further handling, have to be done under the laminar air flow with strict aseptic precautions.

The advantage of having a coating such as gelatin in a petri dish facilitates cell culture right from initial attachment to further multiplication and culture progression. This model was developed to successfully propagate ovarian stem cells while maintaining their characteristics.

However, this study is limited by the absence of a comparative analysis of stemness marker expression between gelatin-coated and commercially available culture dishes. Additionally, cultures were not evaluated beyond the initial passage, limiting insights into long-term effects.

## Conclusions

The isolation and culture of human ovarian stem cells include several critical steps. An ideal culture environment for the initial attachment, culture, and further propagation of cells is possible only with a gelatin-coated dish, as seen in our study. However, the effect of other coating agents, such as laminin and fibronectin, can be studied to identify the ideal component for coating the culture surfaces.
